# Incident Heart Failure in Atherosclerotic Renal Artery Stenosis: A Post Hoc Analysis of the CORAL Trial

**DOI:** 10.1016/j.xkme.2024.100948

**Published:** 2024-12-17

**Authors:** Rajesh Gupta, Michelle M. Estrella, Rebecca Scherzer, Pamela S. Brewster, Lance D. Dworkin, Hanh T. Nguyen, Yanmei Xie, Joachim H. Ix, Michael G. Shlipak, Timothy P. Murphy, Donald E. Cutlip, Eldrin F. Lewis, Christopher J. Cooper

**Affiliations:** 1 Division of Cardiovascular Medicine, University of Toledo College of Medicine and Life Sciences, Toledo, OH; 2 Department of Medicine, University of Toledo College of Medicine and Life Sciences, Toledo, OH; 3 Kidney Health Research Collaborative, San Francisco Veterans Affairs Healthcare System and University of California, San Francisco, CA; 4 Nephrology Section, Veterans Affairs San Diego Healthcare System and Division of Nephrology-Hypertension, University of California, San Diego, CA; 5 Summa Therapeutics, LLC, Cambridge, MA; 6 Beth Israel Deaconess Medical Center and Baim Institute for Clinical Research, Boston, MA; 7 Cardiovascular Division, Stanford University, Stanford, CA

**Keywords:** Albuminuria, cardiorenal syndrome, heart failure, renal artery stenosis

## Abstract

**Rationale & Objective:**

Although renal artery stenosis (RAS) and heart failure (HF) have been linked, the incidence and predictors of HF among patients with RAS are not well described.

**Study Design:**

Post hoc analysis of the Cardiovascular Outcomes in Renal Atherosclerotic Lesions (CORAL) multicenter, open-label, randomized controlled trial (RCT).

**Settings and Participants:**

Patients with atherosclerotic RAS and elevated blood pressure, chronic kidney disease, or both, and without a history of HF at enrollment.

**Intervention:**

Medical therapy alone versus medical therapy plus renal artery stenting.

**Outcomes:**

Incident HF events.

**Results:**

This analysis included 808 participants enrolled in the CORAL trial without evidence of baseline HF. During a median follow-up of 4.8 years, 54 participants (6.7%) developed incident HF. HF incidence rates did not differ by randomized intervention (HR, 0.84; 95% confidence interval [CI], 0.49-1.43 for stent arm with medical arm as reference). Baseline diabetes (subdistribution hazard ratio (sHR), 2.07; 95% CI, 1.20-3.58), albuminuria (sHR, 1.12 per doubling of urinary albumin-creatinine ratio, 95% CI, 1.02-1.24), lower eGFR (sHR, 0.78 per 10 mL/min/1.73 m^2^ estimated glomerular filtration rate calculated with cystatin C and creatinine, 95% CI, 0.69-0.88), and peripheral vascular disease (PVD) (sHR, 2.18, 95% CI, 1.21-3.91) were independent predictors of incident HF. Participants who experienced incident HF had greater kidney function decline before HF events.

**Limitations:**

This is a post hoc analysis of a RCT. The number of HF events is small.

**Conclusions:**

In patients with RAS, rates of incident HF did not differ between participants randomized to optimal medical therapy alone versus optimal medical therapy plus renal artery stenting. The presence of diabetes, PVD, and worse kidney health at baseline were associated with future HF events.

## Plain-Language Summary

Renal artery stenosis has been linked with heart failure. The CORAL randomized controlled trial has the largest study population of participants with renal artery stenosis. In this analysis, we assessed the incidence and predictors of heart failure in CORAL. We found similar rates of incident heart failure among participants randomized to medical therapy alone vs. medical therapy plus renal artery stent. We identified independent predictors of incident heart failure among people with renal artery stenosis include PAD, diabetes, albuminuria, and lower baseline eGFR. In addition, eGFR declined prior to heart failure events.

Renal artery stenosis (RAS) commonly affects patients with other manifestations of atherosclerosis, such as coronary artery disease or peripheral vascular disease (PVD),^1^^,^^2^ and has been associated with high rates of morbidity and mortality.^3^ RAS has been linked to heart failure (HF), with Pickering et al^4^ first describing the association in 1988. Since that time, several randomized controlled trials have investigated the role of RAS and renal artery interventional procedures in the management of hypertension and preservation of kidney function.^5^, ^6^, ^7^ However, the interrelationship between RAS and HF has received less attention.

Observational cohort studies of patients with HF have established that RAS and HF frequently coexist, with an estimated RAS prevalence of 15%-50% in patients with HF.^8^, ^9^, ^10^ Furthermore, RAS is associated with increased rates of adverse events among patients with HF, such as worsening kidney function, recurrent HF hospitalizations, and death.^10^, ^11^, ^12^, ^13^ Nonetheless, the diagnostic and management strategies for preventing HF in patients with RAS remain uncertain.

The Cardiovascular Outcomes in Renal Atherosclerotic Lesions (CORAL) trial was the largest randomized controlled trial of persons with RAS. We sought to determine the incidence of HF among participants in CORAL and the effect of randomization to optimal medical therapy versus optimal medical therapy plus renal artery stent on the rate of incident HF events. Furthermore, we sought to identify baseline predictors of incident HF events.

## Methods

### Study Design and Population

This is a post hoc cohort analysis of data from the CORAL trial (Clinical trial registration: NCT00081731) to assess the incidence of HF, the effect of the trial intervention on HF incidence, and predictors of HF among people with RAS. The CORAL trial was a prospective, international, multicenter clinical trial that compared the effects of optimal medical therapy alone or optimal medical therapy plus renal artery stenting on major cardiovascular or kidney events, defined as a composite of death from cardiovascular or kidney causes, stroke, myocardial infarction, hospitalization for congestive HF, progressive renal insufficiency, or the need for permanent kidney replacement therapy.^7^ Optimal medical therapy comprised treating blood pressure to goal of <140/90 mm Hg in general and to goal of <130/80 mm Hg among participants with diabetes or chronic kidney disease (CKD). Diabetes was treated to a hemoglobin A1c (HbA1c) goal of <7.0%, based on national clinical practice guidelines current at the time of study. Comprehensive medical therapy included use of an angiotensin receptor-blocking drug (candesartan), a calcium channel blocker (amlodipine), a statin (atorvastatin), and antiplatelet therapy (aspirin). Thiazide diuretic was added if blood pressure goal was not met, and further protocol driven blood pressure control was implemented.

A total of 931 participants were assigned in a 1:1 ratio to either medical therapy alone or medical therapy plus renal artery stent. The inclusion criteria required an objectively measured RAS of at least 60%. All angiograms were centrally analyzed by the angiography core laboratory for the study at the University of Virginia. Trial enrollment began on May 16, 2005, and follow-up terminated September 28, 2012. Please see the original publication for details of the inclusion and exclusion criteria.^7^ For the present analysis, we excluded participants with baseline history of HF, resulting in a sample size of 808 participants.

All centers obtained institutional review board (IRB) approval and followed institutional policies and study protocol. The CORAL trial conduct adhered to the Declaration of Helsinki. All participating patients provided written informed consent. The present post hoc analysis of the CORAL clinical trial database was evaluated by the University of Toledo IRB. Because we used a deidentified database, this work is considered nonhuman subjects research and is exempt from IRB review. CORAL was registered with clinicaltrials.gov (NCT00081731).

### Outcomes

The primary outcome for this analysis was incident HF. Hospitalization for congestive HF was an adjudicated trial endpoint, determined by a blinded clinical events committee, and it was defined as being hospitalized for 12 hours or longer because of documented signs and symptoms of HF and treatment with intravenous therapy (vasodilators, diuretics, or inotropes) during the hospital stay. Only the first HF event was counted to determine the number of incident HF events.

### Candidate Covariates

All covariates were obtained at the baseline study visit and included age, gender, race, ethnicity, study arm, body mass index, systolic and diastolic blood pressure, urinary albumin-creatinine ratio (UACR), estimated glomerular filtration rate (eGFR), maximum renal artery percent stenosis, bilateral RAS, smoking within the past year, hyperlipidemia, history of myocardial infarction (MI), transient ischemic attack (TIA)/stroke, PVD, and diabetes mellitus. Hyperlipidemia was defined as total cholesterol >200 mg/dL or treatment with a lipid lowering medication. Diabetes was defined as treatment with oral diabetes medication or insulin or fasting glucose > 126 mg/dL. Bilateral RAS was defined as ≥60% stenosis of bilateral renal arteries. Smoking status, PVD, TIA/stroke, and history of MI were patient reported. UACR was log-transformed to normalize its distribution. We used the CKD Epidemiology Collaboration (CKD EPI) equations to estimate eGFR with cystatin C, creatinine, and the combined equation.^14^

### Statistical Analysis

We summarized demographic and baseline clinical characteristics stratified by intervention arm. We then compared incident HF rates by randomized intervention, baseline eGFR <60 versus ≥60 mL/min/1.73 m^2^, and presence versus absence of baseline albuminuria. Because Kaplan–Meier curves can overestimate the incidence of the outcome over time, we instead plotted cumulative incidence accounting for the competing risk of death. We used Gray's test to compare differences in cumulative incidence by treatment arm.^15^ Multivariable Fine-Gray proportional subhazards regression models were used to assess risk factors for incident HF, evaluating the candidate covariates listed above while accounting for death as a competing risk.^16^ Proportional hazards assumptions were checked by comparing plots of log (−log(survival)) versus log of survival time and the Schoenfeld test. All candidate variables had <10% missingness except for UACR (11%) and stenosis (20%). Multiple imputation with the Markov chain Monte Carlo method for arbitrary missing multivariate normal data was used to impute missing covariates with 10 imputations.

We used Bayesian model averaging^17^ to identify parsimonious sets of risk factors that were independently associated with incident HF, retaining predictors with posterior probabilities >35%. This method accounts for model uncertainty in variable selection by averaging over all candidate models, each weighted by its posterior model probability. As a sensitivity analysis, we stratified the analysis by randomized intervention (Stent + Medical Therapy vs Medical Therapy arm) and tested interactions of each risk factor with the intervention.

Because development of HF may influence the trajectory of eGFR, we used joint models^18^^,^^19^ to simultaneously evaluate repeated measures of eGFR and time-to-HF event data. This approach reduces bias and improves precision over more traditional time-to-event analyses. Our joint model linked 2 sub-models: (1) a linear mixed effect model assessing changes in eGFR values across the baseline and follow-up study visits and (2) an exponential survival model beginning at the baseline study visit. Within the linear mixed effect submodel, we allowed the effect of time on eGFR trajectory to vary by HF status. This process was implemented by including a time-updated HF indicator variable for those who developed HF during the study, such that pre- and post-HF slopes could be estimated. For those who did not develop HF, a separate indicator variable was included to enable this group to have its own slope estimated separately.

Bayesian model averaging was performed using the Bayesian model averaging package from the R statistical computing language (R Development Core Team). All other analyses were conducted using the SAS system, version 9.4 (SAS Institute, Inc).

## Results

**Figure 1:**
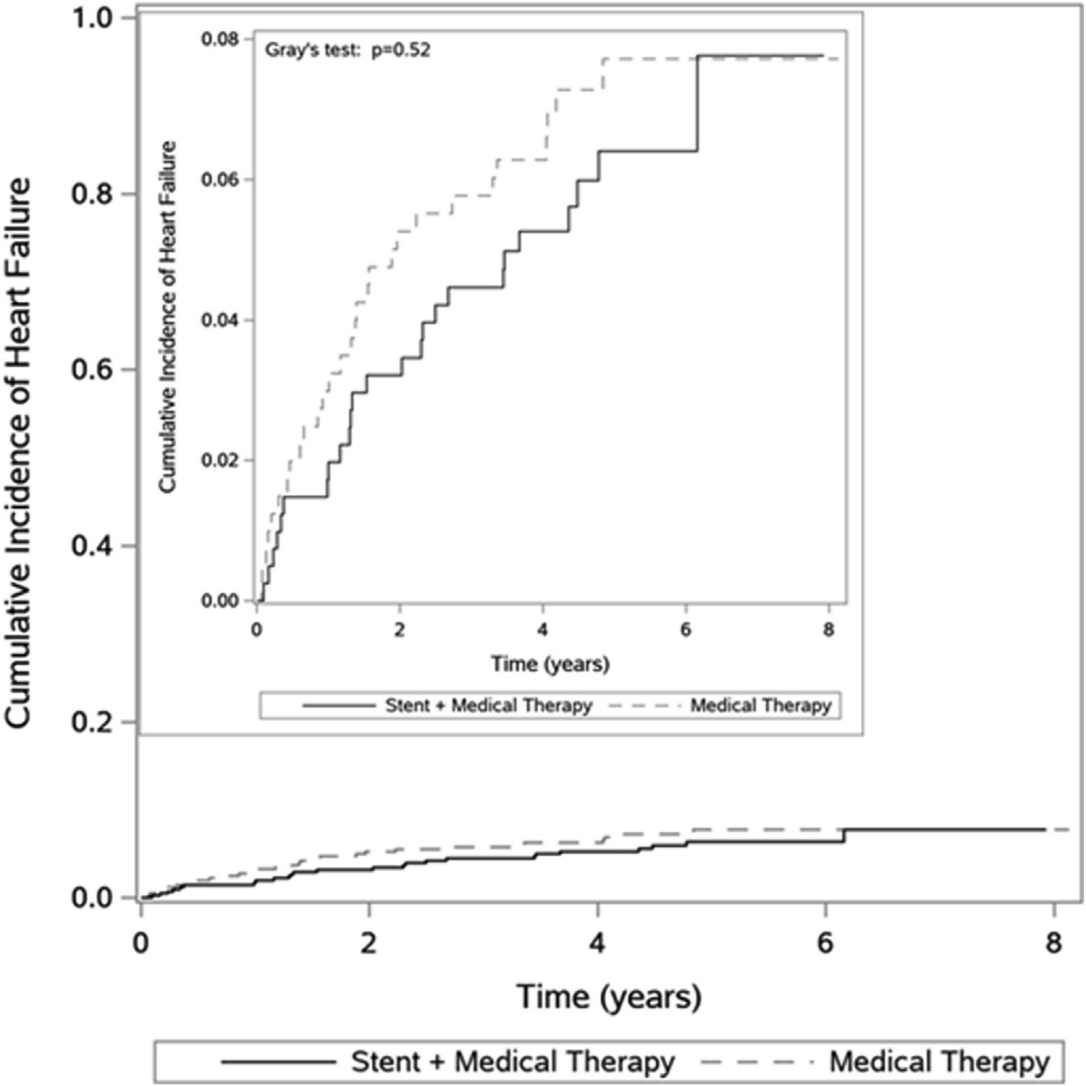
Incidence of HF, stratified by randomization status. Cumulative incidence of HF accounting for competing risk of death. Comparison of differences in cumulative incidence of HF by randomized treatment arm using Gray’s test.

Among the 808 CORAL participants included in this analysis, 54 (6.7%) developed incident HF events during a median follow-up of 4.8 years. Baseline characteristics of participants are shown in Table 1; no differences were observed between the 2 trial arms among this subset of CORAL selected for absence of HF at baseline. There was no difference in the incidence of HF among participants randomized to optimal medical therapy versus renal artery stent plus optimal medical therapy (Fig 1).

**Table 1:** Baseline Characteristics in CORAL, After Excluding Those With Prevalent HF^a^

Parameter	% with Missing Values	Overall	Stent + Medical Therapy	Medical Therapy Only
		N = 808	N = 405	N = 403
Age, y	0	70 (64-77)	71 (64-76)	70 (63-77)
Female	0	413 (51%)	202 (50%)	211 (52%)
Race	0			
African American		55 (7%)	27 (7%)	28 (7%)
White		739 (91%)	372 (92%)	367 (91%)
Other		14 (2%)	6 (1%)	8 (2%)
Hispanic	0	45 (6%)	21 (5%)	24 (6%)
BMI (kg/m^2^)	0.4%	28 (26-32)	28 (25-32)	28 (26-32)
Systolic BP (mm Hg)	0.9%	150 (135-165)	150 (135-165)	151 (134-166)
Diastolic BP (mm Hg)	0.9%	79 (71-88)	79 (71-87)	79 (71-88)
Creatinine (mg/dL)	5.1%	1.16 (0.90-1.48)	1.19 (0.91-1.49)	1.14 (0.89-1.46)
Cystatin C (mg/L)	5.0%	1.18 (0.95-1.46)	1.20 (0.96-1.47)	1.15 (0.94-1.45)
eGFRcyscr (race-free equation) (mL/min/1.73 m^2^)	5.1%	60 (45-80)	60 (45-79)	60 (46-80)
UACR, mg/g	11%	21 (9-75)	22 (10-81)	19 (8-64)
Maximum renal artery percent stenosis	20%	68 (60-77)	69 (61-77)	67 (58-77)
Bilateral RAS	20%	146 (23%)	92 (24%)	54 (20%)
Smoking (within the past year)	0	246 (30%)	115 (28%)	131 (33%)
Hyperlipidemia	0.4%	707 (88%)	354 (88%)	353 (88%)
Myocardial infarction	0.1%	202 (25%)	95 (24%)	107 (27%)
TIA/stroke	2.1%	155 (20%)	80 (20%)	75 (19%)
Peripheral vascular disease	0.1%	391 (48%)	196 (49%)	195 (48%)
Diabetes mellitus	1.6%	246 (31%)	121 (30%)	125 (32%)
CKD ≥ Stage III	0%	502 (62%)	252 (62%)	250 (62%)
Diuretic use	1.1%			
Loop		102 (13%)	49 (12%)	53 (13%)
Thiazide		158 (20%)	71 (18%)	87 (22%)
Both		14 (2%)	5 (1%)	9 (2%)
None		525 (66%)	276 (69%)	249 (63%)

Abbreviations: BMI, body mass index; BP, blood pressure; CKD, chronic kidney disease; eGFR, estimated glomerular filtration rate by creatinine and cystatin C; RAS, renal artery stenosis; TIA, transient ischemic attack; UACR, urinary albumin-creatinine ratio.

a Data displayed as N (%) or median [interquartile range].

**Figure 2:**
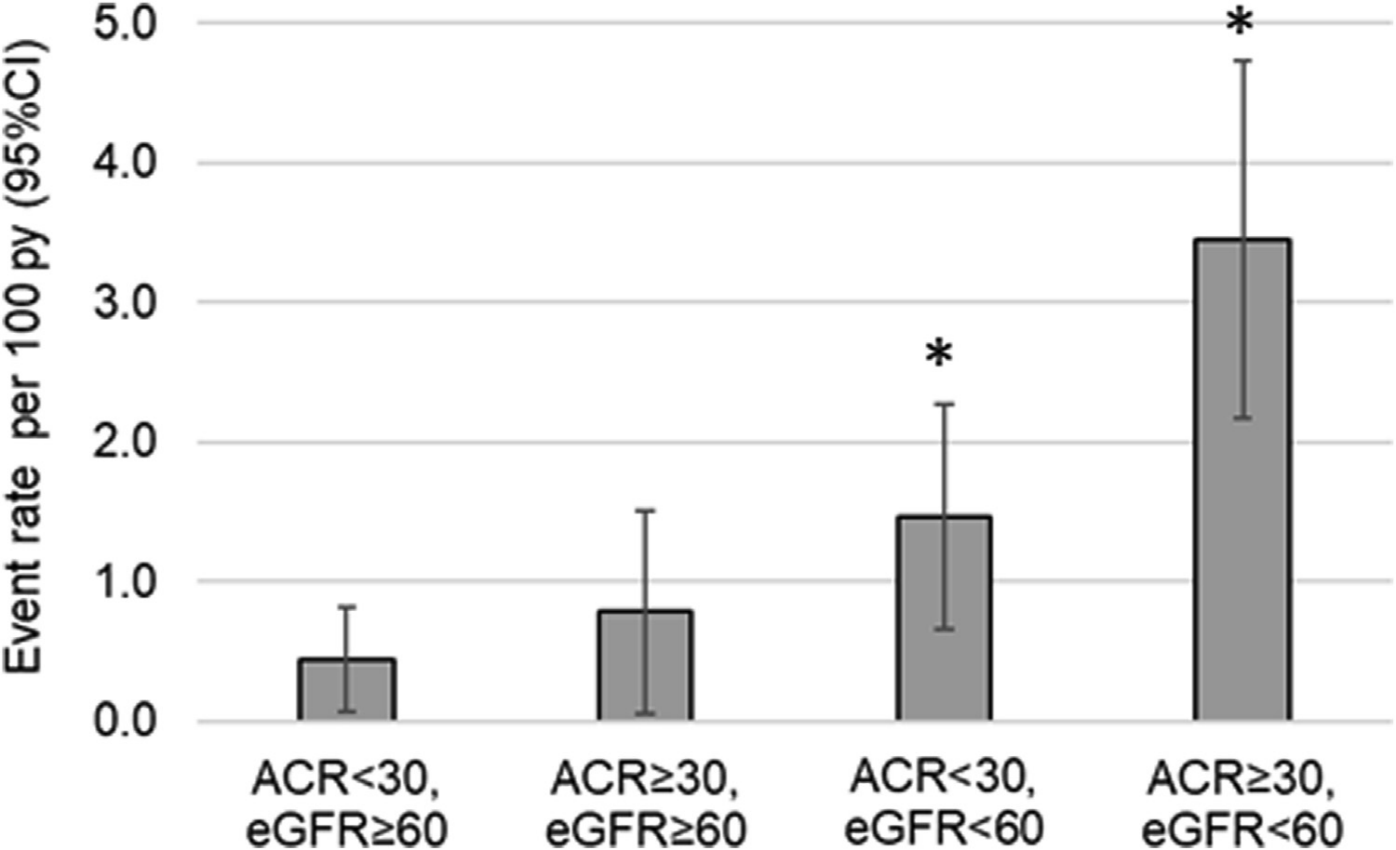
Incident HF event rates by combined categories of baseline eGFRcyscr and UACR. ^a^*P* < 0.05 for comparison with control group (participants with ACR < 30 and eGFR ≥ 60).

In unadjusted analysis, older age, lower eGFR, higher UACR, TIA/stroke, PVD, and diabetes were associated with risk of incident HF. In a multivariable model assessing baseline variables, we identified diabetes, albuminuria, lower eGFR, and PVD as independent variables associated with incident HF (Table 2). We further assessed HF rates by eGFR and UACR categories and found the highest HF rates among participants with eGFR < 60 mL/min/1.73 m^2^ and UACR > 30 mg/g (Fig 2). In stratified analyses, the association of bilateral RAS with incident HF appeared to be modified by randomized intervention, with lower risk of incident HF in the renal artery stent plus medical therapy group (subdistribution hazard ratio [sHR], 0.53, 95% confidence interval [CI], 0.18-1.53) but higher risk in the medical therapy only group (sHR, 1.90; 95% CI, 0.73-4.93); however, the p-interaction of 0.07 did not reach statistical significance. The associations of the remaining baseline factors with incident HF did not appear to be influenced by assigned intervention group (Table 3).

**Table 2:** Associations of Baseline Characteristics With Incident HF in CORAL^a^

Parameter	Unadjusted	Fully adjusted^b^	Parsimonious^c^
	SHR (95% CI)	SHR (95% CI)	SHR (95% CI)
Stent arm	0.84 (0.49-1.43), *P* = 0.52	0.67 (0.36-1.22), *P* = 0.19	0.73 (0.42-1.25), *P* = 0.25
Age (per decade)	1.43 (1.07-1.93), *P* = 0.02	1.24 (0.76-2.02), *P* = 0.38	
Male vs Female	1.45 (0.84-2.48), *P* = 0.18	1.76 (0.97-3.20), *P* = 0.064	
African American vs White	1.40 (0.56-3.49), *P* = 0.47	1.74 (0.65-4.63), *P* = 0.27	
Other vs White	1.11 (0.15-8.25), *P* = 0.92	1.31 (0.17-10.09), *P* = 0.80	
Hispanic	n/a – too sparse to estimate		
BMI (per kg/m^2^)	0.98 (0.94-1.03), *P* = 0.51	0.97 (0.91-1.03), *P* = 0.33	
Systolic BP (per 10 mm Hg)	1.09 (0.97-1.21), *P* = 0.15	1.05 (0.91-1.21), *P* = 0.52	
Diastolic BP (per 10 mm Hg)	0.89 (0.73-1.09), *P* = 0.27	0.85 (0.64-1.13), *P* = 0.27	
eGFRcyscr (per 10 mL/min/1.73 m^2^)	0.74 (0.66-0.83), *P* < 0.001	0.77 (0.67-0.89), *P* = 0.001	0.78 (0.69-0.89), *P* < 0.001
UACR (per doubling)	1.25 (1.14-1.37), *P* < 0.001	1.14 (1.02-1.28), *P* = 0.03	1.13 (1.02-1.25), *P* = 0.02
Max renal artery % stenosis	1.05 (0.80-1.39), *P* = 0.73	1.01 (0.74-1.37), *P* = 0.97	
Bilateral RAS	1.15 (0.59-2.22), *P* = 0.68	1.02 (0.48-2.17), *P* = 0.95	
Smoking (within past year)	0.83 (0.45-1.51), *P* = 0.53	1.05 (0.49-2.27), *P* = 0.89	
Hyperlipidemia	1.20 (0.47-3.05), *P* = 0.70	0.77 (0.29-2.09), *P* = 0.61	
Myocardial infarction	1.47 (0.83-2.60), *P* = 0.18	1.29 (0.70-2.41), *P* = 0.42	
TIA/stroke	1.89 (1.04-3.41), *P* = 0.04	1.42 (0.76-2.66), *P* = 0.28	
Peripheral vascular disease	2.32 (1.29-4.15), *P* = 0.005	1.93 (1.04-3.57), *P* = 0.04	2.23 (1.23-4.02), *P* = 0.008
Diabetes mellitus	2.41 (1.40-4.13), *P* = 0.001	2.19 (1.16-4.13), *P* = 0.02	2.03 (1.17-3.52), *P* = 0.01

Abbreviations: BMI, body mass index; BP, blood pressure; eGFR, estimated glomerular filtration rate by creatinine and cystatin C; RAS, renal artery stenosis; SHR, subdistribution hazard ratio; TIA, transient ischemic attack; UACR, urinary albumin-creatinine ratio.

a Hazard ratios with 95% confidence intervals from Fine-Gray competing risk models.

b Fully adjusted model controls for all risk factors simultaneously.

c Parsimonious model controls for reduced set of risk factors selected by Bayesian Model Averaging, with stent arm forced into the model.

**Table 3:** Associations Of Baseline Characteristics With Incident HF In CORAL, Stratified By Randomized Intervention Group^a^

Parameter	Stent + Medical Rx	Medical Rx Only	Test for Interaction
	SHR (95% CI)	SHR (95% CI)	*P* Value
Age (per decade)	1.04 (0.55-1.97), *P* = 0.91	1.47 (0.85-2.54), *P* = 0.17	0.34
Male vs Female	1.49 (0.64-3.48), *P* = 0.36	2.03 (0.91-4.53), *P* = 0.084	0.60
African American vs White	0.83 (0.12-5.56), *P* = 0.8	2.40 (0.78-7.38), *P* = 0.13	0.34
Other vs White	n/a – too sparse to estimate		
BMI (per kg/m^2^)	0.98 (0.91-1.05), *P* = 0.59	0.96 (0.88-1.05), *P* = 0.39	0.73
Systolic BP (per 10 mm Hg)	1.09 (0.92-1.29), *P* = 0.31	1.01 (0.83-1.22), *P* = 0.93	0.47
Diastolic BP (per 10 mm Hg)	0.97 (0.61-1.54), *P* = 0.90	0.78 (0.59-1.05), *P* = 0.10	0.37
eGFRcyscr (per 10 mL/min/1.73 m^2^)	0.77 (0.63-0.94), *P* = 0.009	0.78 (0.64-0.95), *P* = 0.01	0.92
ACR (per doubling)	1.09 (0.93-1.27), *P* = 0.28	1.20 (1.03-1.41), *P* = 0.02	0.36
Max renal artery % stenosis	1.02 (0.68-1.53), *P* = 0.93	1.00 (0.66-1.50), *P* = 0.99	0.94
Bilateral RAS	0.53 (0.18-1.53), *P* = 0.24	1.90 (0.73-4.93), *P* = 0.19	0.07
Smoking (within past year)	0.76 (0.24-2.35), *P* = 0.63	1.37 (0.55-3.39), *P* = 0.50	0.38
Hyperlipidemia	0.43 (0.12-1.51), *P* = 0.19	1.30 (0.28-6.09), *P* = 0.74	0.27
Myocardial infarction	0.95 (0.40-2.26), *P* = 0.90	1.70 (0.73-3.98), *P* = 0.22	0.34
TIA/stroke	2.08 (0.93-4.68), *P* = 0.08	0.97 (0.36-2.56), *P* = 0.94	0.23
Peripheral vascular disease	1.30 (0.55-3.10), *P* = 0.55	2.72 (1.14-6.47), *P* = 0.02	0.24
Diabetes mellitus	2.10 (0.94-4.73), *P* = 0.07	2.27 (0.95-5.41), *P* = 0.07	0.90

Abbreviations: BMI, body mass index; BP, blood pressure; eGFR, estimated glomerular filtration rate by creatinine and cystatin C; RAS, renal artery stenosis; SHR, subdistribution hazard ratio; TIA, transient ischemic attack; UACR, urinary albumin-creatinine ratio.

a Hazard ratios with 95% confidence intervals from Fine-Gray competing risk models. All estimates above are from fully adjusted models, controlling for all risk factors simultaneously.

Next, we assessed changes in eGFR during follow-up. Participants who experienced incident HF events had greater declines in eGFR compared with participants without HF events, and these declines occurred before HF events (Table 4).

**Table 4:** Estimated Annual Changes in eGFR (mL/min/1.73 m^2^) Among Participants With and Without Incident HF^a^^,^^b^

	Annual change in eGFRcr (95%CI)	*P* value
Slope in those with No HF	–1.5 (–1.7 to –1.2)	*P* < 0.001
Pre-event slope in those with HF	–3.6 (–5.1 to –2.2)	*P* < 0.001
Post-event slope in those with HF	–1.4 (–2.9 to +0.2)	*P* = 0.09

a Estimates obtained from joint models of survival and longitudinal eGFR.

b Tests for differences: pre vs post HF: *P* = 0.003; no HF vs any HF: *P* = 0.01.

In a sensitivity analysis, we assessed the entire CORAL cohort (N = 931, including participants with baseline history of HF). We found similar results overall. The rate of incident HF did not differ among participants randomized to medical therapy versus medical therapy plus renal artery stent (Figure S1). Predictors of incident HF remained similar with estimated glomerular filtration rate by creatinine and cystatin C (eGFRcyscr), UACR, and diabetes being independent predictors. In the new model using the entire CORAL cohort, PVD was no longer significant, and age was a significant predictor of incident HF (Tables S1 and S2).

## Discussion

Among participants with RAS in the CORAL trial, the incidence of HF did not differ when patients were randomized to medical therapy plus renal artery stent versus medical therapy alone. Among persons with RAS, we found that factors independently associated with incident HF included diabetes, albuminuria, lower eGFR, and PVD, and participants who developed incident HF events had greater declines in eGFR before HF events than following the diagnosis of HF.

To our knowledge, our study represents the largest report of the incidence and predictors of HF among persons with RAS. The relationship between RAS and HF has been known for many years, but high-quality data on this topic are limited. We found an approximately 7% (1.4% per year) incidence of HF in CORAL during study follow-up. This is higher than other contemporary clinical trials of individuals with hypertension, such as the SPRINT trial, which reported HF incidences of approximately 2.1% (0.64% per year).^20^ Participants in CORAL had higher baseline systolic blood pressure and lower eGFR compared with the participants enrolled in SPRINT, and SPRINT also excluded persons with diabetes. These factors may explain the higher HF incidence in CORAL.

We found a similar incidence of HF in CORAL regardless of whether participants were randomized to optimal medical therapy versus renal artery stent plus optimal medical therapy. These findings do not support renal artery stent treatment for prevention of HF. Additionally, these findings support the robust medical treatment in the CORAL trial which achieved excellent blood pressure control with high rates of treatment with angiotensin-converting enzyme inhibitors (ACE-I), angiotensin II receptor blockers (ARBs), calcium channel blockers, and thiazide diuretics. The presence of diabetes, albuminuria, lower eGFR, and PVD at baseline were independently associated with incident HF events. Diabetes and albuminuria reflect multifactorial kidney disease in participants with RAS, which may further predispose to HF events. Persons with RAS who have concomitant diabetes and albuminuria may fair especially poorly. In a prior CORAL manuscript, albuminuria was an independent predictor of the composite CKD outcome, which was defined as progression to end stage kidney disease, >30% decline in eGFR, or death caused by kidney disease.^21^ In addition, participants in CORAL with greater than the median levels of albuminuria (UACR > 22.5 mg/g) had higher risk of cardiovascular outcomes during study follow-up.^22^ In the present analysis, the combination of UACR ≥ 30 mg/g and eGFR < 60 mL/min/1.73 m^2^ was associated with a substantially higher rate of incident HF. In fact, there was a 7.6-fold increase in the rate of incident HF when comparing participants with UACR ≥ 30 mg/g and eGFR < 60 mL/min/1.73 m^2^ versus those with UACR< 30 mg/g and eGFR ≥ 60 mL/min/1.73 m^2^ (Fig 2). The presence of lower extremity PVD in this RAS cohort represents a marker for higher total atherosclerosis burden. Prior studies have found that polyvascular disease (atherosclerosis in more than 1 major vascular territory) is associated with higher rates of HF and death compared with atherosclerosis in a single vascular territory.^23^, ^24^, ^25^

Guidelines on RAS and HF give a class I recommendation for revascularization among patients with hemodynamically significant RAS and recurrent, unexplained HF or flash pulmonary edema but highlight the limited high-quality data among patients with concomitant RAS and HF.^26^^,^^27^ Based on the findings from the current analysis, efforts should be made to optimize medical therapy for diabetes and albuminuria to reduce HF events in RAS populations. Future studies should investigate the safety and efficacy of renal protective medical therapies such as SGLT2 inhibitors and mineralocorticoid receptor antagonists for patients with RAS. A multi-targeted medical intervention of ACE-I/ARB, SGLT2 inhibitors, and mineralocorticoid receptor antagonists may potentially provide strong protection against HF events in patients with RAS.

Our study has several limitations that should be noted. This is a post hoc subgroup analysis of a randomized controlled trial. The number of participants that developed incident HF events is small. Echocardiographic data were not ascertained in the CORAL trial, so we cannot compare baseline ejection fraction. The bilateral RAS subgroup was small and the subgroup analysis for the effect of randomized treatment on incident HF may have been underpowered.

In conclusion, the rates of incident HF in the CORAL trial did not differ by randomization status. In patients with RAS, the presence of diabetes, PVD, albuminuria, and lower eGFR at baseline were each independently associated with incident HF. Evaluating trajectories of kidney function, eGFR tended to decline more before rather than after incident HF events in this population of hypertensive trial participants with RAS.
