# Supplementary material for: Incident Heart Failure in Atherosclerotic Renal Artery Stenosis: A Post Hoc Analysis of the CORAL Trial
Source: Kidney Med. 2024 Dec 17;7(2):100948. doi: 10.1016/j.xkme.2024.100948 (PMC11787008; doi:10.1016/j.xkme.2024.100948)
Supplement: Supplementary File (PDF) — Figure S1; Tables S1-S2. [file mmc1.pdf]

Figure S1: Incidence of HF, stratified by randomization status, including participants with and without prevalent HF

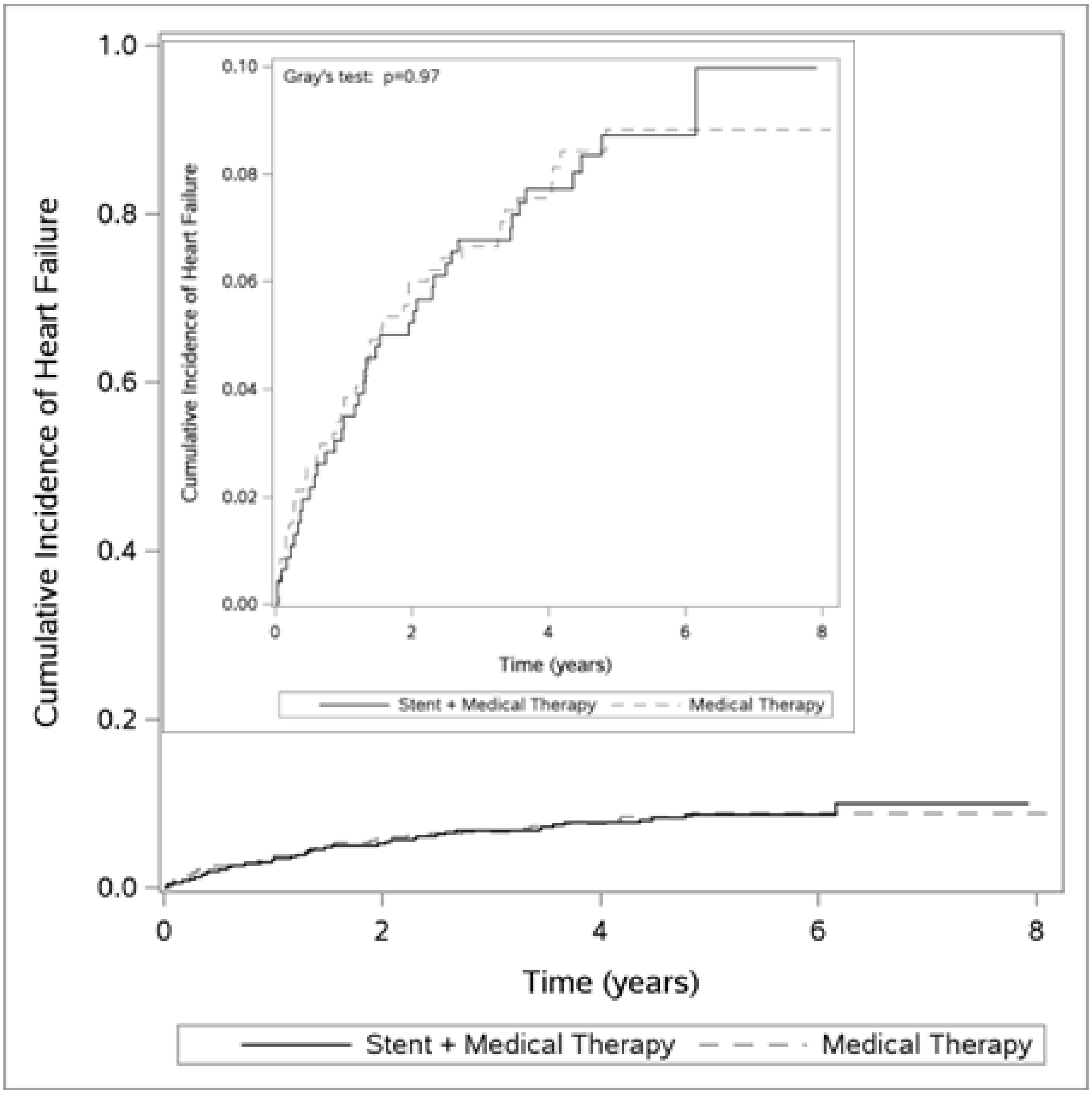

Cumulative incidence of HF accounting for competing risk of death. Comparison of differences in cumulative incidence of HF by randomized treatment arm using Gray's test.

Supplemental Table 1. Baseline characteristics in CORAL, including those with and without prevalent HF

Parameter	Overall N=931	Stent + Medical Therapy N=459	Medical Therapy Only N=472
Age, years	71 (64, 76)	71 (64, 76)	70 (63, 77)
Female	476 (51%)	231 (50%)	245 (52%)
Race			
Black	66 (7%)	32 (7%)	34 (7%)
White	849 (91%)	420 (92%)	429 (91%)
Other	16 (2%)	7 (2%)	9 (2%)
Hispanic	57 (6%)	25 (5%)	32 (7%)
BMI (kg/m^2)	28 (26, 32)	28 (26, 32)	29 (26, 32)
Systolic BP (mmHg)	149 (134, 164)	149 (134, 164)	149 (133, 164)
Diastolic BP (mmHg)	79 (70, 87)	79 (71, 87)	78 (70, 88)
Creatinine (mg/dL)	1.17 (0.91, 1.51)	1.19 (0.91, 1.52)	1.16 (0.90, 1.51)
Cystatin C (mg/L)	1.20 (0.96, 1.50)	1.21 (0.97, 1.52)	1.19 (0.95, 1.50)
eGFRcys-cr (race-free equation) (mL/min/1.73 m^2)	59 (44, 79)	59 (44, 79)	59 (44, 79)
UACR, mg/g	22 (10, 82)	24 (10, 98)	21 (9, 75)
Maximum renal artery percent stenosis	68 (60, 77)	69 (61, 77)	67 (59, 77)
Bilateral RAS	175 (24%)	109 (25%)	66 (21%)
Smoking (within the past year)	277 (30%)	127 (28%)	150 (32%)
Hyperlipidemia	822 (89%)	403 (88%)	419 (89%)
Myocardial infarction	260 (28%)	120 (26%)	140 (30%)
TIA/stroke	181 (20%)	90 (20%)	91 (20%)
Peripheral vascular disease	464 (50%)	226 (49%)	238 (50%)
Diabetes mellitus	308 (34%)	147 (32%)	161 (35%)
CKD≥ Stage III	585 (63%)	286 (62%)	299 (63%)
Diuretic use			
Loop	144 (16%)	69 (15%)	75 (16%)
Thiazide	170 (18%)	76 (17%)	94 (20%)
Both	21 (2%)	8 (2%)	13 (3%)
None	586 (64%)	302 (66%)	284 (61%)

Data displayed as N (%) or median [interquartile range]

Abbreviations: BMI, body mass index; BP, blood pressure; eGFR, estimated glomerular filtration rate by creatinine and cystatin C; UACR, urine albumin-to-creatinine ratio; CKD, chronic kidney disease; TIA, transient ischemic attack; RAS, renal artery stenosis

Supplemental Table 2: Associations of baseline characteristics with incident HF in CORAL, including those with and without prevalent HF

**Parameter**	**Unadjusted SHR (95%CI)**	**Fully adjusted* SHR (95%CI)**	**Parsimonious** SHR (95%CI)**
Stent arm	1.01 (0.65, 1.58), p=0.95	0.85 (0.52, 1.41), p=0.54	
Age (per decade)	1.57 (1.23, 2.00), p=0.001	1.47 (0.98, 2.19), p=0.06	1.41 (1.05, 1.89), p=0.02
Male vs. Female	1.18 (0.76, 1.84), p=0.46	1.59 (0.98, 2.58), p=0.06	
Black vs. White	1.27 (0.58, 2.76), p=0.55	1.62 (0.69, 3.82), p=0.27	
Other vs. White	0.75 (0.10, 5.49), p=0.78	1.00 (0.13, 7.83), p=1.00	
Hispanic	n/a – too sparse to estimate		
BMI (per kg/m^2)	1.00 (0.97, 1.04), p=0.96	0.99 (0.95, 1.04), p=0.80	
SBP (per 10mmHg)	1.03 (0.93, 1.14), p=0.60	0.99 (0.87, 1.12), p=0.84	
DBP (per 10mmHg)	0.84 (0.71, 0.99), p=0.04	0.90 (0.72, 1.12), p=0.33	
eGFRcyscr (per 10 $ml/min/1.73m^2$)	0.71 (0.64, 0.78), p<0.001	0.75 (0.66, 0.86), p<0.001	0.77 (0.69, 0.86), p<0.001
UACR (per doubling)	1.26 (1.17, 1.37), p<0.001	1.16 (1.05, 1.28), p=0.003	1.15 (1.06, 1.25), p=0.001
Max renal artery % stenosis	1.03 (0.82, 1.28), p=0.82	0.94 (0.73, 1.20), p=0.62	
Bilateral RAS	1.33 (0.79, 2.25), p=0.29	1.17 (0.66, 2.09), p=0.59	
Smoking (within past year)	0.71 (0.42, 1.21), p=0.21	1.12 (0.56, 2.23), p=0.76	
Hyperlipidemia	1.18 (0.54, 2.60), p=0.68	0.89 (0.38, 2.07), p=0.78	
Myocardial infarction	1.57 (0.99, 2.48), p=0.05	1.45 (0.87, 2.41), p=0.15	
TIA/stroke	1.63 (1.00, 2.67), p=0.05	1.35 (0.79, 2.30), p=0.28	
Peripheral vascular disease	1.69 (1.06, 2.68), p=0.03	1.41 (0.83, 2.39), p=0.21	
Diabetes mellitus	2.32 (1.48, 3.64), p<0.001	1.85 (1.09, 3.16), p=0.02	1.98 (1.25, 3.13), p=0.004

Hazard ratios with 95% confidence intervals from Fine-Gray competing risk models. SHR = subdistribution hazard ratio
* fully adjusted model controls for all risk factors simultaneously
** parsimonious model controls for reduced set of risk factors selected by Bayesian Model Averaging
